# Effect of Centrifugation of Stallion Semen Through a Low Density Colloid Prior to Freezing on Sperm Cryosurvival

**DOI:** 10.3390/ani15131881

**Published:** 2025-06-25

**Authors:** Ziyad Al-Kass, Jane M. Morrell, Theodoros Ntallaris

**Affiliations:** 1 Department of Surgery and Theriogenology, College of Veterinary Medicine, University of Mosul, Mosul 41002, Iraq; ziyad.al.kass@slu.se; 2 Department of Clinical Sciences, Swedish University of Agricultural Sciences, P.O. Box 7054, SE-75007 Uppsala, Sweden; theodoros.ntallaris@slu.se

**Keywords:** sperm quality, horse, semen, CASA

## Abstract

Sperm quality is adversely affected by cryopreservation due to the increased production of reactive oxygen species, which affects the integrity of sperm membranes, motility, and DNA fragmentation. Three methods for removing seminal plasma, washing (centrifuging extended semen at 800× *g* for 10 min) and Single Layer Centrifugation with high or low density Equicoll, were used to prepare 29 ejaculates from ten stallions for freezing. Sperm quality parameters (kinematics, plasma membrane integrity, superoxide and hydrogen peroxide production, mitochondrial membrane potential, and DNA fragmentation) were evaluated before and after freezing using kinematic and flow cytometric analysis. The parameters for fresh samples were within the normal range for stallion semen but were lower after thawing. There were few differences between the three preparation methods. Interestingly, DNA fragmentation was affected most by the sperm preparation method, being lowest for SLC through high density Equicoll, although SLC through low density Equicoll was effective for some stallions. Some differences were observed in the proportions of live or dead spermatozoa positive for hydrogen peroxide. In conclusion, all of these methods would be suitable for the preparation of semen prior to cryopreservation, but Single Layer Centrifugation through high density Equicoll was the most effective in removing spermatozoa with damaged DNA.

## Simple Summary

There are still challenges in achieving good results when freezing stallion spermatozoa for artificial insemination. The first step in the procedure involves the removal of most of the seminal plasma by centrifugation. Finding a gentler method of removing the seminal plasma could result in better sperm survival during freezing. The purpose of this study was to compare the effect of the usual washing procedure against colloid centrifugation using either a high- or a low density colloid before freezing. Sperm quality was analysed before and after freezing. There were considerable differences in the number of spermatozoa recovered from the three treatments, with the low density colloid producing the most. Some differences in post-thaw sperm quality were seen, notably that DNA damage was less in the method involving centrifugation through a high density colloid. In addition, more metabolic byproducts were produced from the washing treatment, which can have a detrimental effect on sperm quality. Therefore, any of these methods could be used to prepare stallion sperm for freezing, but colloid centrifugation may have some advantages over washing.

## 1. Introduction

In equine breeding, the use of artificial insemination (AI) is growing rapidly, but fresh or cooled semen is preferred to frozen semen because of a perceived reduction in sperm quality in post-thawed samples [1,2]. Moreover, the deposition of semen relative to the time of ovulation is critical when frozen semen is inseminated [3]. Individual variation [4], breed variation [5], and semen collection time [6] are some important factors affecting the use of frozen semen. Cryopreservation has a negative effect by reducing sperm longevity and increasing oxidative damage [3]. Moreover, approximately 20% post-thaw sperm viability was lost when the sample was incubated at 37 °C in a water bath for one hour [7].

Sperm cryopreservation has several advantages over the use of liquid semen, such as increased sperm storage time [8], the genetic improvement of breeding animals [9], and reduced disease transmission [10]. However, cryopreservation causes the destruction and damage of spermatozoa by exposure to oxidative stress, cold, and osmotic shock [11]. Seminal plasma has a deleterious effect on stallion spermatozoa after thawing [12]; therefore, removing seminal plasma before cryopreservation is important, allowing the sperm concentration to be increased.

Several methods are available for removing seminal plasma, but the most commonly used method is to “wash” the spermatozoa, i.e., to add an extender to the semen and then pellet the spermatozoa by centrifugation, thus permitting the removal of most of the supernatant. Other methods have been reported, such as cushion centrifugation, colloid centrifugation, especially with one layer of colloid [13,14], or modified flotation density gradient centrifugation [15].

After centrifugation and aspirating the seminal plasma, a cryoextender is added, and the extended sample is packed into straws. The semen is either frozen directly from the ambient temperature in the vapour above the surface of liquid nitrogen or is allowed to cool slowly to 4 °C for several hours before freezing. In the latter case, freezing can be achieved either using a programmable freezer [16], producing a controlled rate of cooling, or in liquid nitrogen vapour.

Previously, Single Layer Centrifugation (SLC) through a high density colloid was used to separate robust spermatozoa capable of surviving freezing from the rest of the sperm sample [14]. However, some good-quality spermatozoa may be lost along with the poor-quality ones [17]. A recent modification of SLC used a colloid of low density instead of a high-density colloid to remove the seminal plasma without any selection for robust spermatozoa. Such preparation typically allows approximately 85% of the spermatozoa to be retrieved without affecting sperm quality [18].

The purpose of the present study was to compare the three previously mentioned methods of removing seminal plasma from stallion spermatozoa prior to freezing, namely, sperm washing, SLC with high density Equicoll, and SLC with low- density Equicoll. Sperm quality in the thawed samples was analysed by flow cytometry and motility assessment.

## 2. Materials and Methods

**Figure 1:**
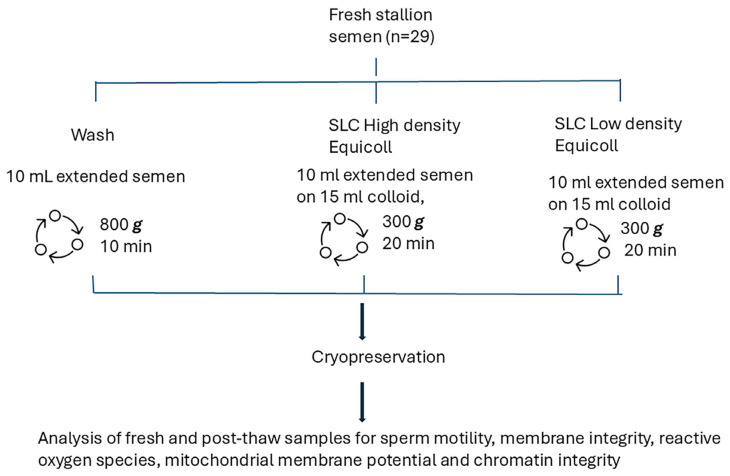
Experimental design comparing three methods of spermatozoa preparation: sperm washing and Single Layer Centrifugation through either high density Equicoll or low density Equicoll (*n* = 29 ejaculates).

Figure 1 shows the experimental design. In brief, sperm kinematics, membrane integrity, superoxide and hydrogen peroxide production, mitochondrial potential, and sperm chromatin structure were analysed in fresh and thawed samples collected from 10 stallions (29 ejaculates).

### 2.1. Animals

Ejaculates were collected from ten warmblood stallions, aged between 5 and 22 years, at a commercial stud in Sweden. The stallions were those available for semen collection for artificial insemination at the time of the study; they were not chosen specifically for the “freezability” of their semen. In total, 29 ejaculates were collected: 3 ejaculates each from 9 stallions and 2 ejaculates from one stallion. The stallions were cared for according to international and national regulations appropriate for the housing and care of breeding animals.

### 2.2. Semen Collection

A Missouri model artificial vagina was used for semen collection. Warm (37 °C) Kenney’s extender was added 1:1 (*v*/*v*) to the semen immediately after collection. Samples were taken to the laboratory at the Swedish University of Agricultural Science (SLU) packed in a Styrofoam box (approximately one–two hours transit time) for further preparation and analysis.

### 2.3. Sperm Concentration

The sperm concentration was measured immediately after arrival at the laboratory using a Nucleocounter SP-100 (Chemometec, Allerød, Denmark), and 5 mL of the reagent S100 (Chemometec, Allerød, Denmark) was added to 50 µL of the semen sample. The mixture was loaded into a cassette containing propidium iodide (PI) before measuring the fluorescence from propidium iodide bound to sperm DNA, hence calculating the sperm concentration [19].

### 2.4. Semen Preparation

After adjusting the sperm concentration to 100 × 10^6^ spermatozoa/mL with Kenney’s extender, each sample was split into three portions. One 10 mL aliquot (wash method—control) was centrifuged at 800× *g* for 10 min using a centrifuge with a swing-out rotor. The second portion was used for SLC with high density Equicoll by pipetting 10 mL of the extended semen over 15 mL of Equicoll without mixing and centrifuging at 300× *g* for 20 min (high) using a centrifuge with a swing-out rotor. The third portion was centrifuged through low density Equicoll (low) using the same protocol as for high. After centrifugation, the supernatant was removed from all samples and the sperm pellet was resuspended in a freezing extender (EquiPlus Freeze 1-Step; Minitube International, Tiefenbach, Germany) to achieve a sperm concentration of 200 × 10^6^. After manually filling and heat-sealing 0.5 mL straws (CRYO-VET France), the sperm samples were placed in a cold bench for 2 h to cool from 18 °C to 5 °C. 

### 2.5. Semen Cryopreservation

The straws were transferred from the cooling bench to the automatic cryopreservation machine (Cryochamber; Cryologic, Australia), where the semen was cooled at 2.5 °C/min from 5 °C to −125 °C. The straws were then plunged into liquid nitrogen and stored until required for analysis.

### 2.6. Thawing

Thawing was performed for 30 s in a water bath at 37 °C.

### 2.7. Semen Analysis

Sperm quality in fresh and thawed samples was analysed as follows:

#### 2.7.1. Computer Assisted Sperm Analysis (CASA)

Sperm motility parameters and kinematics (total motility (TM%), progressive motility (PM%), lateral head displacement (ALH, µm), beat cross frequency (BCF, Hz), straight line velocity (VSL, µm/s), curvilinear velocity (VCL, µm/s), the velocity of the average path (VAP, µm/s) and the ratio straightness (STR), linearity (LIN), and wobble (WOB) were analysed using AndroVision^®^ (Minitüb Abfull und Laborteknik, Germany) and a negative phase contrast microscope (Olympus BX-51; Olympus, Japan). The temperature of the microscope stage was set at 38 °C.

Sperm concentration was adjusted with the appropriate extender, equilibrated to the same temperature as the sample, to achieve a sperm concentration of 20–50 × 10^6^, according to the manufacturer’s instructions for this instrument. After incubating the adjusted fresh and thawed samples for 5 min at 37 °C, a 5 µL drop was placed on a warm glass slide and covered with an 18 × 18 mm cover slip. At least 1000 spermatozoa from a minimum of four fields were evaluated to calculate the sperm motility parameters. The following settings were used for the stallion sperm: ALH < 4.0 and BCF < 4.0 for immotile spermatozoa; VCL < 40.0 and VSL < 10.0 for locally motile spermatozoa; a radius > 10.0 but an R < 60.0 and rotation > 0.70 for spermatozoa with circular motility; and VCL < 120.0 for spermatozoa with slow motility. The frame rate was 60 Hz. 

#### 2.7.2. Flow Cytometry (FC)

Analysis of fresh and thawed samples was carried out with a FACSVerse™ flow cytometer (Becton Dickinson (BD) Biosciences, BD and Company, San Jose, CA, USA). Fluorescence was activated with blue and violet lasers (488 nm and 405 nm, respectively). Detection was performed with the following filters: red (700/32 nm), green (527/32 nm), orange (586/42 nm), and blue (528/45 nm). For each assay, 30,000 cells were analysed, apart from the Sperm Chromatin Structure Assay, where 10,000 cells were evaluated. The proportions of the relevant sub-populations were calculated using FCS Express 5 software (De Novo, Glendale, CA, USA) after gating out the debris.

#### 2.7.3. Reactive Oxygen Species (ROS)

To determine ROS production, staining of the sperm samples was performed with 40 mM Hoechst 33258 (Sigma, Stockholm, Sweden) (HO), 2 mM 20,70 dichlorodihydrofluorescein diacetate (DCFDA), and 40 mM Hydroethidine (Invitrogen, Thermo Fisher Scientific, Eugene, OR, USA) (HE). After diluting all samples in Cell WASH (Becton Dickinson, CA, USA) to achieve a concentration of 2 × 10^6^ spermatozoa/mL, 300 µL aliquots were stained with 3 µL HO, 3 µL HE, and 3 µL DCFDA and incubated for 30 min at 37 °C. After gentle mixing, the samples were analysed by FC. Debris was gated out on the dot plot, and spermatozoa were classified as live or dead superoxide-positive and -negative, and live or dead hydrogen peroxide-positive and -negative [20].

#### 2.7.4. Membrane Integrity (MI)

All samples (300 µL) were adjusted to a concentration of 2 × 10^6^ spermatozoa/mL in Cell WASH (Becton Dickinson, CA, USA) before staining using a LIVE/DEAD™ Sperm Viability Kit (Invitrogen, Waltham, MA, USA), using 0.6 µL of 0.02 µM SYBR^®^14 and 3 µL of 12 µM propidium iodide. Samples were incubated at 37 °C for 10 min and analysed using FC [19]. Sperm with damaged cell membranes fluoresce red, while those with intact membranes fluoresce green.

#### 2.7.5. Sperm Chromatin Structure Assay 

Fresh and thawed samples were prepared by adding 50 µL of each sample at a concentration of 2 × 10^6^ sperm/mL to 50 µL of 0.01 M Tris-HCL, 0.15 M sodium chloride, and 1 mM EDTA (a TNE buffer solution). The mixture was frozen in liquid nitrogen at −196 °C and stored at −80 °C until analysed. Frozen samples were thawed on ice, and 10 µL was mixed with 90 µL of the TNE buffer. The sperm membranes were denatured by adding 200 µL acid detergent solution and, after 30 s, were stained with 600 µL acridine orange (AO). The samples were analysed by FC within 5 min of adding the AO to measure the proportion of spermatozoa with single-strand breaks [21]. 

#### 2.7.6. Mitochondrial Membrane Potential

To evaluate sperm mitochondria, the dye JC-1 (1.5μM of 5,5,6,6-tetrachloro-1,1,3,3-tetraethylbenzimidazolylcarbocyanine iodide; Mitochondrial Membrane Potential Probe, Thermo Fisher Scientific, Invitrogen, Waltham, MA, USA) was used. One mL of the sample (2 million sperm/mL in Cell WASH) was stained with 0.5 µL of JC 1, incubated at 37 °C for 30 min, and analysed using FC. The spermatozoa were categorized as having a high or low mitochondrial function.

#### 2.7.7. Statistical Analyses

All data analyses were performed in SAS^®^ software v 9.4 (SAS Institute Inc., Cary, NC, USA). The Kolmogorov–Smirnov test was used to check the distribution of variables. The procedures MEANS and SGPLOT were used to calculate the means and standard deviations. Due to deviations from normality, STR and VCL values were log-transformed prior to analysis. Statistical comparisons were performed on the log-transformed data to meet model assumptions. For clarity, results are presented as geometric means ± 95% confidence intervals, which were obtained by back-transforming the log-scale estimates. This approach provides a more accurate representation of central tendency and variability for log-normally distributed data.

PROC MIXED was used to calculate the least squares means (LSM ± SEM), and *p*-values produced by the model were adjusted for multiple post-ANOVA comparisons using the Scheffé adjustment. The model used was:

Y_ijk_ = μ + Treatment_i_ + Ejaculate_j_ + Treatment_i_ × Ejaculate_j_ + (1|Stallionₖ) + e_ijk_

where Y_ijk_ is the dependent variable, μ is the overall mean, Treatment_i_ is the fixed effect of treatment i (i = Wash, High_SLC, Low_SLC), Ejaculate_j_ is the fixed effect of Ejaculate_j_ (j = 1, 2, 3), Treatment_i_ × Ejaculate_j_ is the interaction between Treatment_i_ and Ejaculate_j_, and e_ijk_ is the residual error. The variable Stallion was included as a random effect.

While the main statistical analyses focused on treatment effects across the group, individual stallion responses were also visualized to illustrate biological variability as a demonstration of the variation in response to treatment, which is well recognized in stallion semen cryopreservation.

The correlation coefficients were computed with the CORR procedure.

The *p*-values were compared based on an alpha value of 5%. Differences between 0.05 < *p* ≤ 0.10 were considered to be trends.

## 3. Results

### 3.1. Fresh Samples

The results for total and progressive sperm motility, DNA fragmentation index, and membrane integrity for fresh samples are shown in Table 1.

### 3.2. Sperm Yield

Sperm yield was 69 ± 5.9% for sperm washing, 42 ± 5.9% for high density Equicoll, and 80 ± 5.9% for low density Equicoll. Thus, the highest recovery was with the low-density colloid, and the lowest recovery was with the high density colloid (low versus high, *p* < 0.0001; wash versus high, *p* < 0.01).

### 3.3. Post-Thaw Samples

#### 3.3.1. Sperm Motility

**Figure 2:**
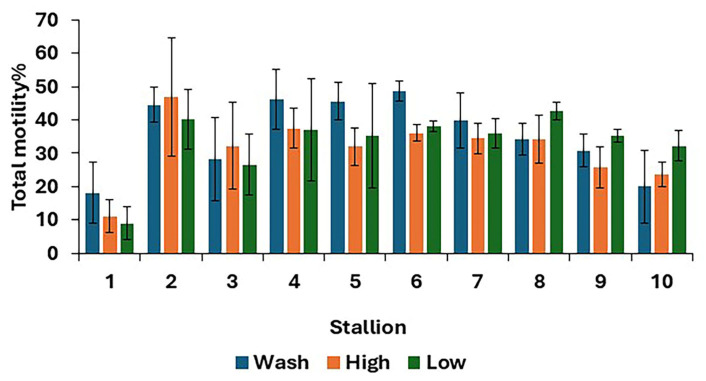
Post-thaw total motility for 10 stallions after freezing in EquiPlus Freeze for wash, high Equicoll, and low Equicoll treatments (least squares means ± standard error; *n* = 29 ejaculates). Note: wash = centrifugation of extended semen at 800× *g* for 10 min; high = Single Layer Centrifugation with high density Equicoll; low = Single Layer Centrifugation with low density Equicoll.

For post-thaw semen samples, no differences (*p* > 0.05) were found in overall means for all sperm kinematics between the three treatments (Table 2). However, there were some differences between treatments in the TM at the individual stallion level (Figure 2).

#### 3.3.2. Reactive Oxygen Species

The different categories of ROS status in post-thaw samples are shown in Table 3. There was a difference (*p* < 0.0001) between wash (1.3 ± 0.1) and high (0.7 ± 0.1) and between wash (1.3 ± 0.1) and low (0.6 ± 0.1) for Live H_2_O_2_ + and between wash (1.4 ± 0.1) and low (0.7 ± 0.1) and (*p* < 0.05) between wash (1.4 ± 0.1) and high (0.87 ± 0.1) for Dead H_2_O_2_+, while there was no difference between treatments for other ROS parameters (Table 3).

#### 3.3.3. Membrane Integrity (MI)

**Figure 3:**
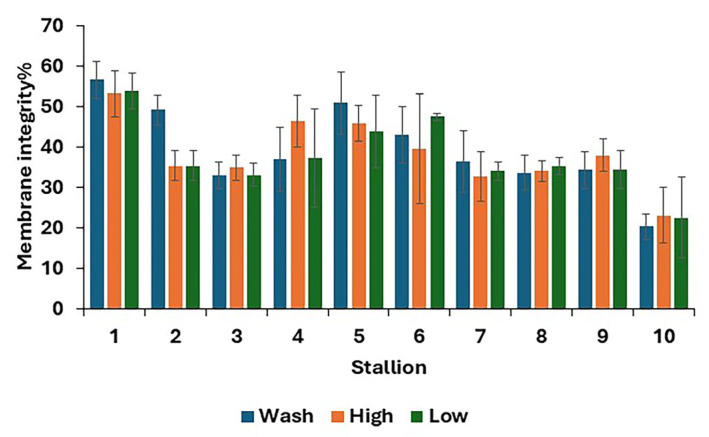
Post-thaw membrane integrity, i.e., stallion spermatozoa with an intact membrane (mean values for each stallion ± standard error) after removing the seminal plasma using different protocols and freezing in EquiPlus Freeze (*n* = 29 ejaculates). Note: wash = centrifugation of extended semen at 800× *g* for 10 min; high = Single Llayer Centrifugation with high density Equicoll; low = Single Layer Centrifugation with low density Equicoll.

For post-thaw samples, there was no difference between treatments in the proportions of living, dead, and dying spermatozoa (Table 4). The results for individual stallions are shown in Figure 3.

#### 3.3.4. The DNA Fragmentation Index (%DFI)

**Figure 4:**
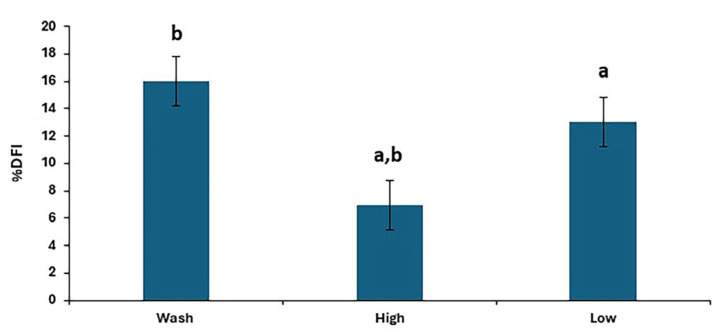
The post-thaw DNA fragmentation index in stallion sperm samples after removing seminal plasma using different sperm preparation protocols and freezing in EquiPlus Freeze. Values are least squares means ± standard error (*n* = 29 ejaculates). Note: wash = centrifugation of extended semen at 800× *g* for 10 min; high = Single Layer Centrifugation with high density Equicoll; low = Single Layer Centrifugation with low density Equicoll. Similar superscript letters refer to a significant difference: ^a,b^ *p* < 0.0001.

**Figure 5:**
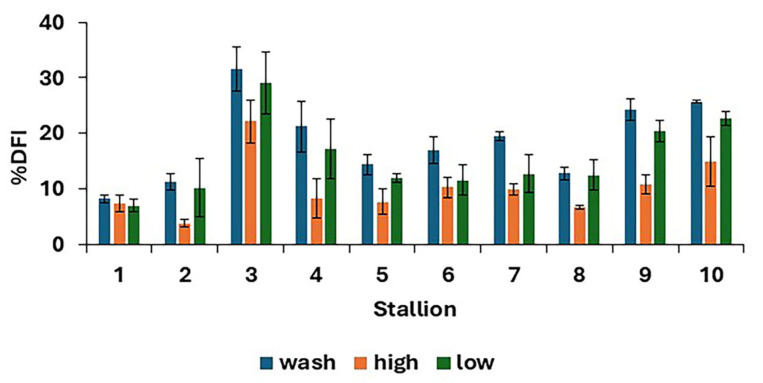
Post-thaw DNA fragmentation index (mean values for each stallion ± standard error) after removing seminal plasma using different protocols and freezing in EquiPlus Freeze (*n* = 29 ejaculates). Note: wash = centrifugation of extended semen at 800× *g* for 10 min; high = Single Layer Centrifugation with high density Equicoll; low = Single Layer Centrifugation with low density Equicoll.

There were differences in %DFI post-thaw between treatments (*p* < 0.0001), as shown in Figure 4, with the results for individual stallions and treatments shown in Figure 5. The proportion of spermatozoa with single-strand DNA breaks was highest for the washed samples and lowest for the high. However, the low resulted in a lower %DFI than sperm washing for some stallions.

#### 3.3.5. Mitochondrial Membrane Potential

**Figure 6:**
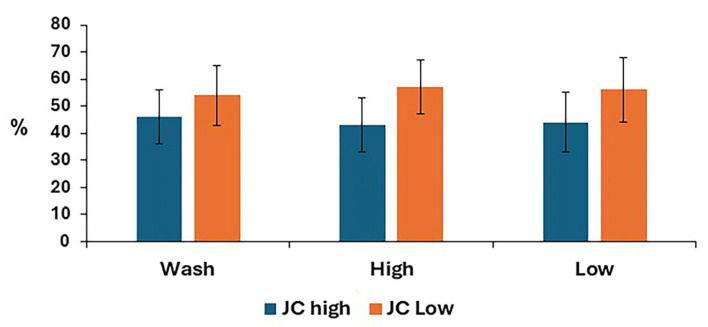
Mitochondrial membrane potential in stallion sperm samples after removing seminal plasma using different sperm preparation protocols and freezing in EquiPlus Freeze. Values are least squares means ± standard error (*n* = 29 ejaculates). Note: wash = centrifugation of extended semen at 800× *g* for 10 min; high = Singl Layer Centrifugation with high density Equicoll; low = Single Layer Cwith low density Equicoll.

The mitochondrial membrane potential for post-thaw stallion sperm samples is shown in Figure 6. There was no difference between treatments for either low or high mitochondrial membrane potential.

#### 3.3.6. Correlation

Negative correlations were seen between %DFI (R^2^ = −0.5, *p* < 0.0001) and MI, between MI and JC low (R^2^ = −0.4, *p* < 0.001), and between living sperm and %DFI (R2 = −0.5, *p* < 0.0001) (Table 5). Positive correlations were observed between MI and JC high (R^2^ = 0.4, *p* < 0.001) and between TM and PM (R^2^ = 0.9, *p* < 0.0001).

## 4. Discussion

The objective was to compare different methods for removing seminal plasma from stallion semen prior to freezing, namely, sperm washing, SLC with high density Equicoll, and SLC with low density Equicoll, for their effect on post-thaw sperm quality. Our hypothesis was that a gentler method of removing the seminal plasma might be beneficial in terms of post-thaw sperm quality. Previous studies showed that colloid centrifugation could improve cryosurvival compared to sperm washing [14,22], although sperm losses were high. Reducing the colloid density might enable more sperm to be recovered, which proved to be the case in the present study: sperm recovery was higher with low density SLC compared with wash and high density SLC.

The quality of the sperm samples on arrival at the laboratory was within the expected ranges for stallion semen collected for artificial insemination. In general, total motility and membrane integrity should be >70% [23,24]. There was a marked effect of the preparation method on post-thaw DNA fragmentation, live H_2_O_2_-positive spermatozoa, and dead H_2_O_2_-positive spermatozoa. Single Layer Centrifugation with low density Equicoll resulted in lower production of ROS than the other preparation methods. Other ROS categories did not differ in overall mean post-thaw values, although sometimes, there were differences between individuals. The cryopreservation of stallion semen affected sperm motility, viability, mitochondrial functions, and ROS production in agreement with other studies (e.g., [25]).

The range of post-thaw motilities in the samples observed here is in agreement with previous studies, where post-thaw motility varied from 20 to 61% [14,26,27,28]. However, PM was higher in their studies than in the present study. This could be due to the cryopreservation protocol used or to the settings on the CASA, which make comparisons between different studies difficult [29]. Progressive motility in post-thaw samples was not affected by the preparation method, in contrast to previous results [22].

A programmed freezing machine was used, which theoretically should have allowed a rapid cooling rate from 4 °C to −125 °C. However, in practice, the machine cooled down more slowly than stated in the program, which may have impacted the sperm quality [30,31]. In addition, the type of cryopreservation extender used can have a considerable effect on post-thaw sperm parameters [32].

Cryopreservation is a stressful method leading to an increase in the production of ROS [33]; reducing ROS production could prevent cryopreservation-induced sperm damage [34]. Our results show significant differences between treatments for the proportions of live hydrogen peroxide-positive and -negative spermatozoa. Both high- and low density SLC resulted in fewer live H_2_O_2_+ compared to washing, while low density SLC also showed fewer dead H_2_O_2_+ compared to both washing and high-density SLC. These results are in agreement with Al-Essawe et al. [28].

The DNA fragmentation differed depending on the treatments used, with the high-density colloid producing sperm samples with less DNA fragmentation than the other methods. These results are in agreement with Gutiérrez-Cepeda et al. [35]. However, for some individuals, even SLC with a low-density colloid resulted in post-thaw sperm samples with less DNA fragmentation than washing, which is an important result since increased %DFI can have a negative impact on fertility [36].

The membrane integrity results were in agreement with a previous study where MI varied from 29 to 40% [37], although they were lower than in other studies where MI ranged from 56.6 to 70.3% [14] and between 41 and 53% [38]. However, other studies indicate that the time between collection and cryopreservation affects post-thaw sperm MI [22,39]. In the present study, several hours elapsed between collecting the semen and the start of processing because of travelling from the stud farm, which may have affected the post-thaw sperm quality. The mitochondrial membrane potential was not affected by treatment, as also observed in a previous study [32].

Although post-thaw membrane integrity was apparently not affected by the sperm preparation method, there was a negative correlation between %DFI and MI and between MMP and MI. Therefore, it might have been possible to detect differences between treatments with a larger sample size. Another study also showed a negative correlation between MI and %DFI [40].

The samples for this study came from stallions that were not selected for the freezability of their semen. Freezability varies considerably between stallions; it was reported previously that approximately one-third of stallions produced ejaculates that do not survive freezing [41]. Therefore, it was not surprising that two individuals in the present study had a post-thaw motility of < 30%. However, it was possible to increase the post-thaw motility of the ejaculates from one stallion using SLC. It would be interesting to process more ejaculates thought to be of poor freezability to see if SLC would be beneficial in such cases.

## 5. Conclusions

All three methods for removing seminal plasma were suitable for preparing stallion semen for freezing. However, low density SLC is more promising because it enables more spermatozoa to be recovered and reduces oxidative stress. Furthermore, importantly, the high density SLC produced post-thaw samples with better DNA integrity, although the sperm losses were higher. Interestingly, in this study, there was stallion-to-stallion variation, indicating that no method works best for all. It would be interesting to expand the study to include more stallions of poor freezability.

## Figures and Tables

**Table 1:** Sperm quality in fresh stallion semen in Kenney’s extender (1:1) at 2 h after collection. Values are presented as mean ± standard errors (*n* = 29 ejaculates).

Stallion	TM%	PM%	MI%	%DFI
1	65 ± 10	47 ± 21	84 ± 1.1	13.4 ± 12.6
2	81 ± 6	81 ± 9	88 ± 6.2	8.6 ± 1.2
3	71 ± 4	52 ± 7	70 ± 14.2	30.3 ± 7.8
4	78 ± 2	75 ± 3	80 ± 7	17.6 ± 7.4
5	79 ± 2	74 ± 4	86 ± 6.7	13.7 ± 2.7
6	74 ± 2	67 ± 3	83 ± 7.1	13.9 ± 0.3
7	77 ± 3	76 ± 2	81 ± 2.9	13.8 ± 3
8	76 ± 1	72 ± 2	87 ± 10.7	10.7 ± 2.4
9	75 ± 4	71 ± 6	75 ± 2.6	22.8 ± 1.8
10	72 ± 3	59 ± 13	77 ± 6.2	23.7 ± 5.7

Notes: TM = total motility; PM = progressive motility; MI = membrane integrity; %DFI = DNA fragmentation index.

**Table 2:** Post-thaw sperm motility for wash, high and low Single Layer Centrifugation (*n* = 29 ejaculates; least squares means ± standard error, except for STR and VCL, which are geometric means and confidence intervals after log-transforming and back-transforming data).

Analysis	Variable	Wash	SLC with High-Density Colloid	SLC with Low-Density Colloid
Least squares means ± SE				
	TM%	35 ± 2.3	31 ± 2.3	33 ± 2.3
	PM%	18 ± 1.9	15 ± 1.9	18 ± 1.9
	WOB	0.43 ± 0.008	0.43 ± 0.008	0.45 ± 0.008
	VSL (µm/s)	9 ± 1.2	7.8 ± 1.2	10.4 ± 1.2
	VAP (µm/s)	11 ± 1.2	10 ± 1.2	12 ± 1.2
	LIN	0.33 ± 0.02	0.33 ± 0.02	0.35 ± 0.02
	BCF (Hz)	5.4 ± 0.5	4.8 ± 0.5	5.6 ± 0.5
	ALH (µm/s)	0.4 ± 0.02	0.3 ± 0.02	0.4 ± 0.02
Mean; upper and lower CI				
	VCL (µm/s)	23.98; 20.04–28.69	20.73; 17.28–24.88	25.23; 20.56–30.95
	STR	0.80; 0.77–0.83	0.80; 0.77–0.83	0.83; 0.80–0.85

Note: wash = centrifugation of extended semen at 800× *g* for 10 min; SLC = Single Layer Centrifugation, total motility (TM), progressive motility (PM), curvilinear velocity (VCL), straightness (STR), beat cross frequency (BCF), straight line velocity (VSL), linearity (LIN), wobble (WOB), lateral head displacement (ALH), and velocity of the average path (VAP).

**Table 3:** Reactive oxygen species production in post-thaw stallion sperm samples for wash, high-density Equicoll, and low-density Equicoll (least squares means ± standard error; *n* = 29 ejaculates).

Variable	Wash	High	Low
Live SO+ (%)	13 ± 0.8	14 ± 0.8	13 ± 0.8
Live SO− (%)	47 ± 2	46 ± 2	46 ± 2
Live H_2_O_2_+ (%)	1.3 ± 0.1 ^ab^	0.7 ± 0.1 ^a^	0.6 ± 0.1 ^b^
Live H_2_O_2−_ (%)	59 ± 2.1	61 ± 2.1	60 ± 2.1
Dead SO− (%)	40 ± 2.1	39 ± 2.1	40 ± 2.1
Dead H_2_O_2_+ (%)	1.4 ± 0.1 ^ac^	0.9 ± 0.1 ^c^	0.7 ± 0.1 ^a^
Dead H_2_O_2−_ (%)	39 ± 2.1	39 ± 2.1	40.1 ± 2.1

Note: SO = superoxide, H_2_O_2_ = hydrogen peroxide. Similar superscript letters within a row refer to a significant difference: ^a,b^ *p* < 0.0001, ^c^ *p* < 0.05.

**Table 4:** Membrane integrity of post-thaw stallion sperm samples for wash, Single Layer Centrifugation with high density Equicoll and Single Layer Centrifugation with low density Equicoll. (Least squares means ± standard error; *n* = 29 ejaculates).

Variable	Wash	High	Low
Living	39.3 ± 2.1	38.6 ± 1.8	37.3 ± 1.8
Dead	56.6 ± 2.1	57.9 ± 1.8	59.1 ± 1.8
Dying	2.1 ± 0.2	2.4 ± 0.2	1.8 ± 0.2

Notes: wash = centrifugation of extended semen at 800× *g* for 10 min; high = Single- Layer Centrifugation with high density Equicoll; low = Single Layer Centrifugation with low density Equicoll.

**Table 5:** Associations among sperm motility, membrane integrity, DNA fragmentation index, and mitochondrial membrane potential in thawed stallion sperm samples (*n* = 29).

Variable	TM	MI	%DFI
PM	R^2^ = 0.9, *p* < 0.0001	------------	------------
Living	------------	------------	R^2^ = −0.49286 *p* < 0.0001
%DFI	------------	R^2^ = −0.49286 *p* < 0.0001	------------
JC high	------------	R^2^ = 0.34605 *p* < 0.0010	------------
JC low	------------	R^2^ = −0.36126 *p* < 0.0006	------------

Note: TM = total motility; PM = progressive motility; %DNA = DNA fragmentation index.

## Data Availability

The original contributions presented in this study are included in the article. Further inquiries can be directed to the corresponding author(s).
